# Spectral composition of LED light differentially affects biomass, photosynthesis, nutrient profile, and foliar nitrate accumulation of lettuce grown under various replacement methods of nutrient solution

**DOI:** 10.1002/fsn3.3735

**Published:** 2023-10-10

**Authors:** Hamid Reza Soufi, Hamid Reza Roosta, Foad Fatehi, Mansour Ghorbanpour

**Affiliations:** ^1^ Department of Horticultural Sciences, Faculty of Agriculture Vali‐e‐Asr University of Rafsanjan Rafsanjan Iran; ^2^ Department of Horticultural Sciences, Faculty of Agriculture and Natural Resources Arak University Arak Iran; ^3^ Department of Agriculture Payame Noor University (PNU) Tehran Iran; ^4^ Department of Medicinal Plants, Faculty of Agriculture and Natural Resources Arak University Arak Iran

**Keywords:** lettuce, light emitting diodes, NO_3_^−^ accumulation, nutrient film technique, photosynthesis

## Abstract

To enhance crop yield and quality, plant cultivation in controlled‐growing systems is an alternative to traditional open‐field farming. The use of light‐emitting diode (LED) as an adjustable light source represents a promising approach to improve plant growth, metabolism, and function. The objective of this study was to assess the impact of different light spectra (red, red/blue (3:1), blue, and white) with an emission peak of around 656, 656, 450, and 449 nm, respectively, under various replacement methods of nutrient solution (complete replacement (CR), EC‐based replacement (ECBR), and replacing based on plant needs (RBPN)), on biomass, physiological traits, and macro‐ and micronutrient contents of two best‐known lettuce varieties, Lollo Rossa (LR) and Lollo Bionda (LB), in the nutrient film technique (NFT) hydroponic system. The results indicated that mix of red and blue LED spectra under RBPN method is the most effective treatment to enhance fresh and dry weights of lettuce plants. In addition, red LED spectrum under RBPN, and red and blue light under ECBR nutrient solution significantly increased leaf stomatal conductance, net photosynthesis and transpiration rate, and intercellular CO_2_ concentration of LR variety. Phosphorus (P), potassium (K), calcium (Ca), and magnesium (Mn) content in LR variety, and iron (Fe), zinc (Zn), copper (Cu), and manganese (Mn) content in both varieties increased upon exposure to blue and red LED light spectrum with RBPN method. Our results suggest that exposure to combination of red and blue light along with feeding plants using RBPN and ECBR methods can increase absorption of macro‐ and micronutrient elements and improve photosynthetic properties, and eventually increase lettuce yield with lower nitrate accumulation.

## Graphical abstract

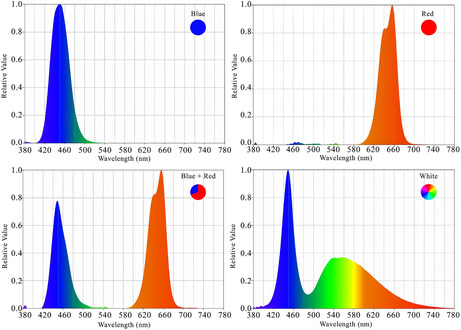

## 1 INTRODUCTION

Hydroponic systems are used to grow leafy vegetables and various plants such as tomatoes, squash cucumbers, peppers, eggplants, strawberries, lettuce, and many others (Barbosa et al., 2015). In the closed cultivation system, the difference in the rate of absorption of water and nutrients, as well as the rapid discharge of some elements from the nutrient solution and the accumulation of some other elements, cause the nutrient and/or ion composition imbalance in the solution (Lopez et al., 2003; Savvas, 2002). The high rate of water absorption compared to nutrients increases the osmotic pressure of the solution and has a negative effect on water absorption, growth, and biomass yield (Samarakoon et al., 2006).

Controlled environment agriculture (CEA) in essence refers to indoor, soil‐less systems where light, temperature, humidity, water, and nutrient availability are carefully controlled, and in recent decades there has been increasing interest in various forms (Goodman & Minner, 2019). This system usually uses artificial LED lighting that can be tailored to selected crops, including leafy greens, cucumbers, tomatoes, strawberries, eggplants, and peppers (Yang & Kim, 2020). The advantage of CEA can be mentioned to avoid soil degradation, including erosion, salinization, and compaction (Atmadja et al., 2017; Khan, 2018), crop losses from extreme weather and various pests and diseases (Yuvaraj & Subramanian, 2020), and nutrient leaching (Oertel et al., 2016). The use of 90% less water to produce 20 times more food than conventional agricultural methods is another advantage of CEA based on hydroponic systems (Lages Barbosa et al., 2015). The nutrient film technique (NFT) is a type of hydroponic system that promotes recirculation and reuse of the nutrient solution and maximizes the water use efficiency in exposed plants (Montesano et al., 2005) that can be used in controlled environment agriculture. Other benefits of NFT systems include production all year round, facilitating cultivation and farming practices, enhancement of the inputs use, control over climate and adverse environmental impacts, and the end products are much cleaner providing more convenience for consumers in cleaning the products and materials before consumption (Lopez et al., 2003).

Replacing the used water automatically prevents the accumulation of elements, but the emptying of the elements must be compensated. It has been recommended that determination of each element concentration should be started during the use of nutrient solution, and according to the amount of absorption of the elements, a filler solution that contains all or some of the elements depleted from the nutrient solution should be added to the initial starter solution (Bugbee, 2004). Savvas (2002) proposed two models for preparation of the additive solution, which was based on the precise monitoring of individual elements in the drained nutrient solution. However, Bugbee (2004) reported that although element monitoring can be very useful, it is not necessary, and instead of depleted nutrients and EC adjustment, and suggested using an additive solution with concentrations lower than the original solution concentration to compensate for the reduction in nutrient solution volume. Application of circulation system and replacement of the nutrient solution every 4 weeks caused an increase in EC and the accumulation of some ions and showed a negative effect on the yield and quality of tomato fruits (Lopez et al., 2003).

Many horticultural plants (such as leafy, root, and fruit vegetables) have been cultivated with hydroponic in a closed growth chamber with a new type of light source such as light‐emitting diode (LED) called plant factory with artificial lighting (PFAL) or vertical farms with artificial lighting (VFALs) (Sabzalian et al., 2014; Zha & Liu, 2018). Manipulation in LED light environment (e.g., establishing specific spectral modes by integrating light quality (spectral composition) and quantity (intensity and photoperiod), and circadian rhythm traits from microsecond to hour levels) can enhance productivity and macro‐ and micronutrient concentration of lettuce crop in a plant factory (Chen & Yang, 2018; Jao & Fang, 2004; Leonel et al., 2023). It has been acknowledged that plants are able to sense the surrounding environment by obtaining information through perceiving light signals in the visible region of the radiation spectrum (400–700 nm) which are the most effective for photosynthesis, photoreceptors, and dynamically absorb and utilize various nutrient elements (Xu et al., 2021). Previous studies indicated that nutrient uptake and utilization by horticultural plants are changed by light parameters including light quality, light intensity, and photoperiod via a complex regulatory network (Chen et al., 2016; Kazemi et al., 2023; Paradiso & Proietti, 2022; Satari et al., 2022). Also, there is a link between light perception and nutrient uptake (Cui et al., 2019; Lin et al., 2020; Zhai et al., 2019). Other studies suggest that red light increases N content, while the blue‐light spectrum is effective in absorption of P and K in Chinese chive (*Allium tuberosum*) plant (Chen, 2012). In pseudo‐stems of garlic (*Allium sativum* L.) seedling, blue light increased the content of N, P, and K (Yang, 2011). Also, previous studies showed that red light had more effect on Fe content, and blue light increased N and Ca and decreased K and Mg in spinach (*Spinacia oleracea* L.) (Qi, 2007). Xu (2007) reported that blue light may improve the absorption of Zn, Fe, Mg, and Cu in the leaf tissue of lettuce (*Lactuca sativa*) plant. According to Miao et al. (2019), red light regulates the uptake and accumulation of P, K, Mn, and Zn in cucumber. It has also been reported that in sprouting broccoli (*Brassica oleacea* var. italica) cultivated under combined red and blue light (R/B = 1: 4), the amount of Ca, Mg, P, S, B, Cu, Fe, Mn, Mo, and Zn were increased (Kopsell et al., 2014). In a study by Degni et al. (2021) on okra (*Abelmoschus esculentus* L.), results showed that red light increased absorption of Ca, P, and Mn, and blue light improved N content. Lettuce grown under recycled hydroponic subjected to combination of light quality (red + blue) increased the amount of nitrate (NO_3_^−^) and nitrite (NO_2_^−^) contents (Li et al., 2021). Continuous irradiation of light on lettuce plants caused a decrease in N, P, K, Ca, Mg, Cu, and Zn nutrients in shoot, while no change was observed in the content of Fe and Mn elements under such conditions (Son et al., 2018). According to Liu et al. (2020), light intensity up to 200 μmol m^−2^ s^−1^ on lettuce plants increases the content of N, P, Ca, Mg, Fe, Mn, Cu, and Zn elements in the shoot of the lettuce plant. Moreover, the use of light combination 70:18:12 (blue: green: red) caused a significant increase in leaf biomass but prevented the accumulation of K and Mg elements in the leaf of lettuce plant (Matysiak et al., 2021). They noted that the multiple combinations of light quality (red‐, green‐, and blue‐light spectrum) in the stages close to lettuce harvest caused a significant decrease in the content of K, P, and NO_3_^−^ content, but the content of Ca showed a significant increase (Matysiak et al., 2021). Exposure to the combination of red and blue lights caused an increase in the content of N, P, K, Ca, Mg, and Fe in basil plants (Pennisi et al., 2019). Furthermore, red LED light increased the amount of K, Ca, Mg, Na, and S in carnation (*Dianthus caryophyllus*) cultivars, while blue LED light was more effective on P content (Manivannan et al., 2017). Amoozgar et al. (2017) indicated that lettuce cv. Grizzly treated with red LED spectrum had the highest amount of Fe, Cu, Mn, and Zn compared to the other light treatments. Also, Liu et al. (2016) reported that combination of red and blue LED lightening improved the amount of NO_3_^−^ in lettuce.

The growth, productivity, and physiological traits of plant are related to light because these parameters are regulated by the intensity, spectra, and time regime of light (Landi et al., 2020; Ptushenko et al., 2020). Researchers believe that analyzing the influence of light spectra on physiological and growth traits during cultivation of different plant species is an important problem (Yudina et al., 2022). The increase in the blue‐light intensity can decrease the biomass, dry weight (DW), light use efficiency, and linear electron flow (LEF), and increase the stomata conductance, cyclic electron flow, dark respiration, and content of chlorophylls and carotenoids (Chen et al., 2021; Yudina et al., 2022), while the increased intensity of the red light induces the opposite effect because physiological processes, growth, and production of lettuce plants are strongly affected by light spectra (Yudina et al., 2023). The use of blue light can increase the respiration rate and cyclic electron flow around photosystem I in comparison to red light; in contrast, blue light can decrease photosynthetic linear electron flow and various plant growth parameters, such as final biomass (Yudina et al., 2022). The use of red and blue LED light increased the rate of net photosynthesis and transpiration in tomato mutants (Vereshchagin et al., 2023). In another study, results showed that interaction of LED irradiance levels and the nutrient solution EC increased the fresh weight of the stem and the dry weight of all the plant parts (leaves, stem, and roots) of lemon basil (Daud et al., 2023). Soltani et al. (2023) suggested that a combination of R and B light is suitable light spectrum to promote plant growth and photosynthetic performance in tomato seedlings. The researchers stated that white and blue LED light can cause significant changes in the amount of photosynthesis characteristics, so white light increases the intensity of photosynthesis and blue light decreases the intensity of photosynthesis in tomato plants (Vereshchagin et al., 2023). Saito et al. (2010) believed that photosynthesis rate of lettuce increased in monochromic red light and the combination of red and blue light.

This experiment was aimed to assess the impact of different light spectra (red, red/blue (3:1), blue, and white) with peaks at 656, 656, 450, and 449 nm, respectively, and different nutrient solution replacement methods (complete replacement (CR), EC‐based replacement (ECBR), and replacing based on plant needs (RBPN)) on yield, physiological traits, and macro‐ and micronutrient contents of two lettuce varieties in the nutrient film technique (NFT) hydroponic system.

## 2 MATERIALS AND METHODS

### 2.1 Plant materials, growth conditions, and experimental setup

This experiment was conducted in a greenhouse of Vali‐e‐Asr University in 2020. The seeds of lettuce varieties (Lollo Rossa (LR) and Lollo Bionda (LB)) were purchased from Sepahan Rooyesh Co, Isfahan (Iran). The seeds were sown into the seed tray filled with fine perlite medium. Normally, after the four‐leaf stage, the seedlings were transplanted inside the small plastic net pot containing horticultural grade perlite medium. These small pots were situated into holes in the NFT plastic channels. Seedlings were transferred to three NFT systems after 21 days of growth using two polyethylene gullies (dimensions: 200 × 20 × 12 cm, respectively, length, width, and height). Then, each of the two gullies was connected to a vertical gully containing a channel for efficient transfer of nutrient solution to the reservoir tank (capacity: 50 L). On each channel, there were 12 holes with a distance of 20 cm from each other. After transferring the plants to the NFT systems, the Resh nutrient solution (EC: 2.1 ds m^−1^, pH: 7) formulated for lettuce was used (Resh, 2022). EC and pH were monitored daily during the cultivation process with portable pH meter (Fisherbrand™ accumet™ AP115 Portable pH Meter Kit) and portable EC meter (HI9033 EC Meter, Setare Arsh Aria company). The prepared nutrient solution was then fed through a submerged pump into the end of gully from a supply tank. This nutrient solution returned to the reservoir tank through gravity flow. Gully inclination and the flow rate were 1% and 2.4 L min^−1^, respectively. The nutrient solution (5 mM KNO_3_, 5 mM Ca(NO_3_)_2_, 2 mM MgSO_4_, 1 mM KH_2_PO_4_, 7 μM MnCl_2_, 0.7 μM ZnSO_4_, 0.8 μM CuSO_4_, 0.8 μM Na_2_MoO_4_, 25 μM Fe‐EDDHA, and 2 μM H_3_BO_3_) was applied to the seedlings immediately after transplanting. Deionized water was used for nutrient preparation. After transplanting (seedling in four leaves stage) of lettuce varieties, nutrient solutions were replaced by three different replacement methods (complete replacement, partial replacement based on EC, and partial replacement according to plant needs) for 40 days.

In complete nutrient solution replacement treatment, the nutrient solution was replaced weekly. For partial replacement according to EC, the EC of the nutrient solution was adjusted to 2.2 dS m^−1^ by adding predetermined amounts of potassium sulfate, calcium nitrate, magnesium sulfate, potassium dihydrogen phosphate, and half strength of micronutrient solution every 48 h. In the replacement treatment based on the needs of the plants, potassium nitrate is used at the same concentration as in the original solution, and the amount of calcium nitrate, magnesium sulfate, and potassium dihydrogen phosphate was reduced by three quarters and the microelements (Fe, Zn, Cu, and Mn) were reduced by half and added every 2 days based on the amount of water added to the plastic container in which the plants were cultivated. Environmental condition in greenhouse was a temperature of 25/15 (day/night), a photoperiod of 16/8 h (day/night), and a relative humidity of 50 ± 5%.

### 2.2 LED tubes and light treatments

**FIGURE 1:**
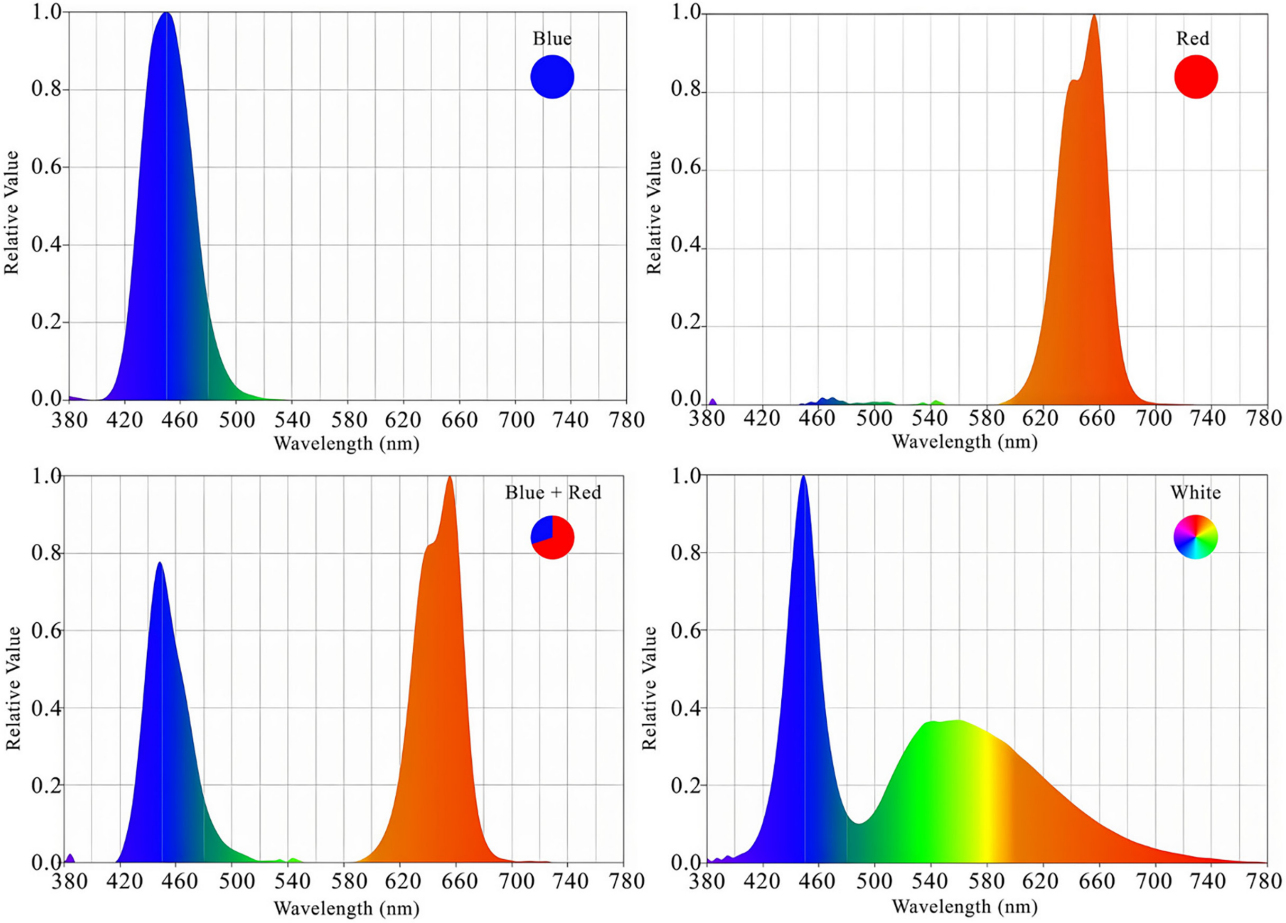
Relative spectral distribution of the different LED (red, red/blue (3:1), blue, and white) used in this study.

Lettuce plants were cultivated under LED lamps with a power of 24 W (Parto Roshd Novin Ltd Co., Iran) at different spectral ranges [red (R, peak at 656 nm), red/blue (3:1) (R: B, peak at 656 nm), blue (B, peak at 450 nm), and white + ambient light (400–700 nm)]. The LED lamps were installed at an appropriate height and distance (30 cm) from the plant canopy to make the optimum and same photosynthetically active radiation (PAR) which was about 215 ± 5.5 μmol m^−2^ s^−1^ for all employed treatments. Details on the characteristics of LEDs used in the present study are given in Table 1 and Figure 1.

**TABLE 1:** Specific features of LEDs used in the present study.

Manufacture company	CRI (color rendering index)	No. of LEDs	Light coverage area	Power consumption	Lens type	Input voltage	DC voltage	Output current	Output frequency
Iran grow light	90%	24	40 × 100 cm	24 × 3 W	90°	AC 100–260 V	54–84 V	600 mA ± 5%	50/60 Hz

### 2.3 Vegetative characteristics

At the end of the experiment (40 days after transplanting), the plants were harvested from each NFT channel and divided into shoots and roots. Dry mass (DM) was obtained when samples were dried in an oven for 72 h at 70°C.

### 2.4 Mineral elements analysis

The aboveground parts of the studied plants were collected for chemical analyses. Collected materials were dried at 45–50°C and then ground to obtain a homogenous size. For determination of nitrogen (N), phosphorus (P), potassium (K), calcium (Ca), magnesium (Mg), and sodium (Na), plant materials were mineralized in concentrated sulfuric acid, while for analysis of total iron (Fe), manganese (Mn), zinc (Zn), and copper (Cu), a mixture of nitric and perchloric acids (3:1, v/v) was used (Estefan et al., 2013). After mineralization of plant materials, the following determinations were performed. Total N: using the distillation method according to Kjeldahl in a Parnas–Wagner apparatus (Kjeldahl, 1883). The value of K, Ca, Mg, Na, Fe, Mn, Zn, and Cu: using atomic absorption spectroscopy (ASA, on a Carl Zeiss Jena apparatus). Also, total phosphorus content was determined using UV–VIS Spectrophotometer T80 (PG Instrument, UK) according to vanadomolybdophosphoric yellow color procedure (Kacar & İnal, 2008).

### 2.5 Nitrate (NO_3_ ^−^) measurement

For NO_3_^−^ analysis, leaf samples of lettuce varieties were chapped and mixed with a food processor. Briefly, 50–100 g of leaves were weighed and placed into a mixer. Distilled water was added to the samples and the mixture was homogenized for 10 min. Tissue homogenate (30 g of sample) was placed in a sterile centrifuge tube, 0.5 mL of hydrogen peroxide (H_2_O_2_) was added, and the tube was capped and mixed well by shaking after each addition. All samples were centrifuged at 3500 rpm at 25°C for 3 min. The supernatant was then separated and filtered through a filter paper (Watchman's no. 1), and NO_3_^−^ concentration in the filtrate was measured colorimetrically by a flow injection analysis technique (Anderson, 1979). The content of NO_3_^−^ was expressed as mg NO_3_^−^per kg on a fresh weight basis (mg NO_3_/kg FW).

### 2.6 Photosynthesis and leaf gas exchange

Photosynthetic and gas exchange parameters were determined using an Infra‐Red Gas Analyzer System (IRGAs, LCi Ultra Compact, ADC BioScientifc Limited, Hertfordshire, UK) (with use of PAR control by LED light: up to 2400 μmol m^−2^ s^−1^ RGB LED array, or up to 2500 m^−2^ s^−1^ by white LED array; CO_2_ concentration: 400 ppm; chamber temperature: 20 ± 3 and precision thermistor ±0.2°C accuracy; H_2_O: 30 mbar and 0.1 mbar resolution; two laser‐trimmed, fast response water vapor sensors: Direct leaf temperature: 25°C self‐positioning microchip thermistor/energy balance/manually positioned thermistor; flow rate to leaf chamber: 68 to 340 μmol m^−2^ s^−1^; and gas connections: 3 mm barbed) following the procedure used by Afridi et al. (2020). Around 9:00 a.m. and 12:00 a.m., measurements were performed on completely expanded leaves. Briefly, one fully expanded and intact leaf was selected from each replicate and clamped to the leaf chamber of the instrument. After 5 min (for warm up at 20°C) and 15 min adaptation to achieve stable steady‐state conditions, net photosynthetic rate (*Pn*; μmol m^−2^ mol^−1^), stomatal conductance (*gs*; mol m^−2^ s^−1^), transpiration rate (*E*; mol H_2_O m^−2^ s^−1^), intercellular CO_2_ concentration (*Ci*; μmol CO_2_ mol^−1^), and intrinsic water‐use efficiency (*P*_N_/*g*s) (μmol CO_2_ mol H_2_O^−1^) were noted.

### 2.7 Statistical analysis

This study was arranged in a three factors factorial experiment based on completely randomized design (CRD) with three replications (*n* = 3). SAS software version 9.4 was used to analyze the obtained data (SA institute, Cary, NC, USA). A two‐way ANOVA model was used to test the statistical analysis of data. Duncan's multiple‐range test (DMRT) was used as a post hoc test to determine the specific differences between group means. Differences were considered significant at *p* ≤ .05. Multivariate analyses of variance were performed using XLSTAT software (Addinsoft, New York, USA). The results were expressed as mean values ± standard errors (SE) of the means.

## 3 RESULTS

### 3.1 Shoot dry mass

Significant differences (*p* ≤ .01) were found for shoot dry mass values between lettuce varieties, replacement methods of nutrient solution, light spectrum, and the interaction of three experimental factors (Table 2). It was also found that the use of RBPN and ECBR methods for both varieties upon exposure to the combination of red/blue LED lights had pronounced effects on shoot dry mass as compared to the other treatments (Table 2).

**TABLE 2:** The impacts of light quality and various employed methods of nutrient solution replacement on shoot and root dry mass weights, and nitrogen content of two lettuce varieties in the NFT system.

Lettuce varieties	Light spectrum	Shoot dry mass (g plant^−1^)			Root dry mass (g plant^−1^)			Nitrogen content (% DW)		
		CR	ECBR	RBPN	CR	ECBR	RBPN	CR	ECBR	RBPN
LR	W	6.10mn	6.89f–k	6.71 h–l	2.23no	2.71h–j	2.45k–n	6.09de	6.10de	5.27h–j
	B	6.40k–m	5.49o	6.20l–n	2.98fg	1.94p	2.36mn	4.87kl	4.88kl	4.21n
	R/B	7.18d–i	7.58c–e	7.9bc	2.88gh	3.16ef	3.57c	7.31a	7.33a	6.32cd
	R	7.32c–g	7.11e–j	5.76no	2.67i–l	2.75hi	3.10ef	5.85ef	5.86ef	5.03jk
LB	W	6.46k–m	6.59i–m	7.24d–h	2.50j–m	2.33mn	3.45cd	6.70b	6.72b	5.79fg
	B	7.54cde	7.75b–d	6.04m–o	3.25de	3.00fg	2.13op	5.36hi	5.37hi	4.63lm
	R/B	7.44c–f	8.27ab	8.62a	2.83gh	4.36b	4.60a	6.40c	6.41c	5.53gh
	R	6.82g–k	6.58i–m	6.53j–m	2.59i–l	2.42l–n	2.45k–n	5.12i–k	5.13i–k	4.42mn

*Note*: Different letters indicate significant differences (*p* ≤ .05) between treatment groups according to Duncan's test.

Abbreviations: B, blue; CR, complete replacement; ECBR, EC‐based replacement; LB, Lollo Bionda; LR, Lollo Rossa; RBPN, replacing based on plant needs; R/B, Red/Blue; W, White.

### 3.2 Root dry mass

The root dry mass was significantly influenced by the employed treatments (*p* ≤ .01, Table 2). According to the results, LB variety fed with RBPN and ECBR methods, and combination treatment of red and blue LED increased root dry mass by 44.36% and 41.28% compared to control (white light and CR method), respectively. Also, LR variety, upon combination of red/blue LED light fed with RBPN and ECBR methods, had the highest root dry mass (Table 2). The root dry mass of LR decreased by 14% under treatment with blue LED light and ECBR method as compared to control (Table 2).

### 3.3 Nitrogen

Foliar N content was significantly influenced by the employed treatments (*p* ≤ .01, Table 2). Also, results showed that the amount of *N* in the CR and ECBR methods in LR variety treated with red/blue LED light increased by 16.91% and 16.68%, respectively, compared to control (withe light spectra). Also, LR showed the minimum amount of *N* when exposed to blue‐light spectra under RBPN method, while in LB, all of the experimental treatments caused a decrease in *N* content over the control (white light and CR method) (Table 2).

### 3.4 Potassium

The K content was significantly (*p* ≤ .01, Table 3) affected between lettuce varieties, replacement methods of nutrient solution, light spectrum, and the interaction of employed factors. Results also indicated that LR exposed to combination of red/blue LED lights fed with RBPN and ECBR methods increased the amount of *K* by 18.5% and 17%, respectively, compared to white light spectra, but in LB, white light spectra under treatment with RBPN showed the highest *K* content (Table 3). Also, the red‐light spectra in LB under RBPN method had the lowest *K* value (Table 3).

**TABLE 3:** The impacts of light quality and various employed methods of nutrient solution replacement on K, Ca, and Mg contents of lettuce varieties in the NFT system.

Lettuce variety	Light spectrum	Potassium (K) (% DW)			Calcium (Ca) (% DW)			Magnesium (Mg) (% DW)		
		CR	ECBR	RBPN	CR	ECBR	RBPN	CR	ECBR	RBPN
LR	W	3.78d	3.72de	3.01jk	1.95f–h	1.90f–i	1.48m–o	1.86g–i	1.92f–h	1.47lm
	B	3.03jk	2.97kl	2.41o	1.56l–n	1.52mn	1.18p	1.48lm	1.57k–m	1.18o
	R/B	3.61ef	4.46a	4.54a	2.03e–g	2.47ab	2.54a	1.96fg	2.41ab	2.55a
	R	3.63ef	3.57f	2.89l	1.93f–h	1.98e–h	1.54l–n	1.93f–h	2.04ef	1.53lm
LB	W	3.31g	4.09b	4.16b	1.63k–m	2.09ef	1.78h–k	2.04ef	1.62j–l	1.77h–j
	B	3.33g	3.27gh	2.65m	1.87g–i	1.82h–j	1.42no	1.78g–j	1.88f–i	1.41mn
	R/B	3.97c	3.9c	3.16hi	2.15de	2.28cd	2.34bc	2.23cd	2.15de	2.35bc
	R	3.18hi	3.12ij	2.53n	1.72i–l	1.67j–m	1.30po	1.63j–l	1.72i–k	1.53lm

*Note*: Different letters indicate significant differences (*p* ≤ .05) between treatment groups according to Duncan's test.

Abbreviations: B, blue; CR, complete replacement; ECBR, EC‐based replacement; LB, Lollo Bionda; LR, Lollo Rossa; RBPN, replacing based on plant needs; R/B, Red/Blue; W, White.

### 3.5 Calcium

The effects of replacement techniques of nutrient solution, light spectrum, varieties, and their interactions were significant on foliar Ca content (*p* ≤ .01, Table 3). It was observed that the combination of red/blue LED lights in LR increased the Ca concentration in plants fed with RBPN and ECBR methods by 23.2% and 21%, respectively, over the white LED light (control) (Table 3), as well as in LB, application of RBPN and ECBR methods increased Ca content under combination of red/blue‐lights spectra. However, the use of blue‐light spectra in LR decreased the concentration of Ca by 25%, 28.2%, and 65.2% in CR, ECBR, and RBPN methods compared to white light spectra, respectively (Table 3).

### 3.6 Magnesium

Foliar Mg content was significantly (*p* ≤ .01) influenced by the employed treatments (Table 3). Results showed that exposure to blue‐light spectra in LR decreased the concentration of Mg compared to white light spectra by 25.6%, 18.4%, and 69% in CR, ECBR, and RBPN methods, respectively (Table 3). Moreover, Mg concentration increased by combination of red/blue‐light spectra in all three replacement methods as compared to white light spectra (control) in both varieties (Table 3).

### 3.7 Phosphorous

The effects of replacement methods of nutrient solution, light spectrum, type of varieties, and the interaction of these factors were significant on P content (*p* ≤ .01, Table 4). Results revealed that in LR, combination of red/blue‐ and red‐light spectra under RBPN and combination of red/blue upon ECBR methods increased P content by 26.2%, 20.8%, and 20.8%, respectively, compared to white light spectra. Also, in LB, combination of red/blue had the maximum effect on P content under RBPN method. Moreover, the use of red‐light spectra in LB fed with RBPN decreased the P content of shoots when compared to the other employed light spectra (Table 4).

**TABLE 4:** The impacts of light quality and various employed methods of nutrient solution replacement on P, NO_3_^−^, and Fe contents of lettuce varieties in the NFT system.

Lettuce varieties	Light spectrum	Phosphorus (% DW)			Nitrate (NO_3_^−^) (mg kg^−1^ DW)			Iron (Fe) (mg kg^−1^ DW)		
		CR	ECBR	RBPN	CR	ECBR	RBPN	CR	ECBR	RBPN
LR	W	0.79e	0.74f	0.52k	2040b	1448d	1064fg	111.4g–i	105.8ij	88.2mn
	B	0.73f	0.69g	0.48k	2346a	1665c	1224e	105.8ij	100.5jk	83.8n
	R/B	0.62h	0.96b	1.03a	1428d	1013g	745h	114.7f–h	137.6b	144.9a
	R	0.62h	0.89c	0.96b	1642c	1165ef	856h	109.0hi	130.7bc	138.5b
LB	W	0.57i	0.89c	0.62h	2102b	1491d	1096fg	133.7bc	127.0cd	105.9ij
	B	0.81de	0.83d	0.58i	2061a	1462d	1075fg	120.6d–f	120.6d–f	100.6jk
	R/B	0.87c	0.88c	0.95b	1471d	1044fg	767h	122.6de	116.4f–h	97.0kl
	R	0.81de	0.76f	0.53j	1442d	1023g	752h	116.4e–g	110.6g–i	92.2lm

*Note*: Different letters indicate significant differences (*p* ≤ .05) between treatment groups according to Duncan's test.

Abbreviations: B, blue; CR, complete replacement; ECBR, EC‐based replacement; LB, Lollo Bionda; LR, Lollo Rossa; RBPN, replacing based on plant needs; R/B, Red/Blue; W, White.

### 3.8 Nitrate

The effects of employed replacement techniques of nutrient solution, light spectrum, type of varieties, and their interactions were significant (*p* ≤ .01) on foliar NO_3_^−^ content (Table 4). Results showed that the blue and white LED lights in both varieties upon all three replacement techniques of nutrient solution increased NO_3_^−^ content (Table 5), and combination of red/blue and monochromic red LED light led to decrease in NO_3_^−^ level of both varieties fed with all three replacement procedures of nutrient solution (Table 4).

**TABLE 5:** The impacts of light quality and various employed methods of nutrient solution replacement on Cu, Zn, and Mn contents of lettuce varieties in the NFT system.

Lettuce varieties	Light spectrum	Copper (Cu) (mg kg^−1^ DW)			Zinc (Zn) (mg kg^−1^ DW)			Manganese (Mn) (mg kg^−1^ DW)		
		CR	ECBR	RBPN	CR	ECBR	RBPN	CR	ECBR	RBPN
LR	W	52.5d	46.9e	33.3i	46.4e–g	44.5hi	34.6n	13.8cd	12.5e	10.2h
	B	47.8e	42.7f	30.3j	42.6jk	40.9l	31.8o	12.4e	11.3f	9.25i
	R/B	43.3f	61.0b	68.2a	41.6kl	53.4b	55.6a	12.3e	15.1b	16.6a
	R	39.4g	55.5c	62.1b	38.2m	49.1d	51.2c	11.1fg	13.6d	14.9b
LB	W	63.0b	56.3c	40g	35.7n	45.8f–h	38.1m	15.2b	13.8cd	11.3f
	B	57.3c	51.2d	36.4h	46.9ef	45.0g–i	35.0n	13.7cd	12.4e	10.1h
	R/B	57.7c	51.6d	36.6h	47.7de	48.9d	51.0c	14.2c	12.9e	10.5gh
	R	52.5d	46.9e	33.3i	43.9ij	42.1kl	32.8o	12.8e	11.6f	9.5i

*Note*: Different letters indicate significant differences (*p* ≤ .05) between treatment groups according to Duncan's test.

Abbreviations: B, blue; CR, complete replacement; ECBR, EC‐based replacement; LB, Lollo Bionda; LR, Lollo Rossa; RBPN, replacing based on plant needs; R/B, Red/Blue; W, White.

### 3.9 Iron

The effects of replacement methods of nutrient solution, light spectrum exposure, lettuce varieties, and the interaction of these factors were significant on foliar Fe content (*p* ≤ .01, Table 4). According to the results, combination of red/blue lights and monochromic LED in LR variety fed with RBPN method increased foliar Fe content by 23.1% and 19.5%, compared to white light spectra (control), while in LB variety, all light spectra under three employed replacement methods decreased the amount of Fe compared to control light (Table 4).

### 3.10 Copper

Foliar Cu content was differentially affected by replacement of the nutrient solution strategies, light spectrum, varieties, and their interactions (*p* ≤ .01, Table 5). As can be observed, the combination of red/blue and monochromic LED lights in LR variety fed with RBPN increased the foliar Cu content by 23% and 15.4% compared to white light spectra (control), while in LB variety, all spectra under three employed replacement methods decreased the amount on Cu percentage over the white control light (Table 5).

### 3.11 Zinc

The Zn content was significantly (*p* ≤ .01) influenced by the employed treatments (Table 5). Also, the combination of red/blue and monochromic red LED lights in LR variety fed with RBPN increased foliar Zn level by 16.5% and 13% over the control (white LED), respectively, however, in LB, combination of red/blue and LED lights led to an increase in foliar Zn content under all three replacement methods (Table 5). The lowest Zn content was observed in LR fed with RBPN method exposed to blue‐light spectra (Table 5).

### 3.12 Manganese

There were significant effects among the replacement methods of nutrient solution, light spectrum, lettuce varieties, and interactions of these factors on foliar Mn content (*p* ≤ .01, Table 5). As results showed, RBPN increased the Mn content of LR variety upon combination of red/blue and monochromic red LED lights by 16.8% and 7.38%, while LR plants fed with ECBR method under combination of red and blue light improved Mn content by 8.6% compared to the control (white LED light) (Table 5). In LB, all light spectra decreased foliar Mn content under three replacement procedures of nutrient solution over the white light spectra (Table 5).

### 3.13 Net photosynthesis rate (a)

**FIGURE 2:**
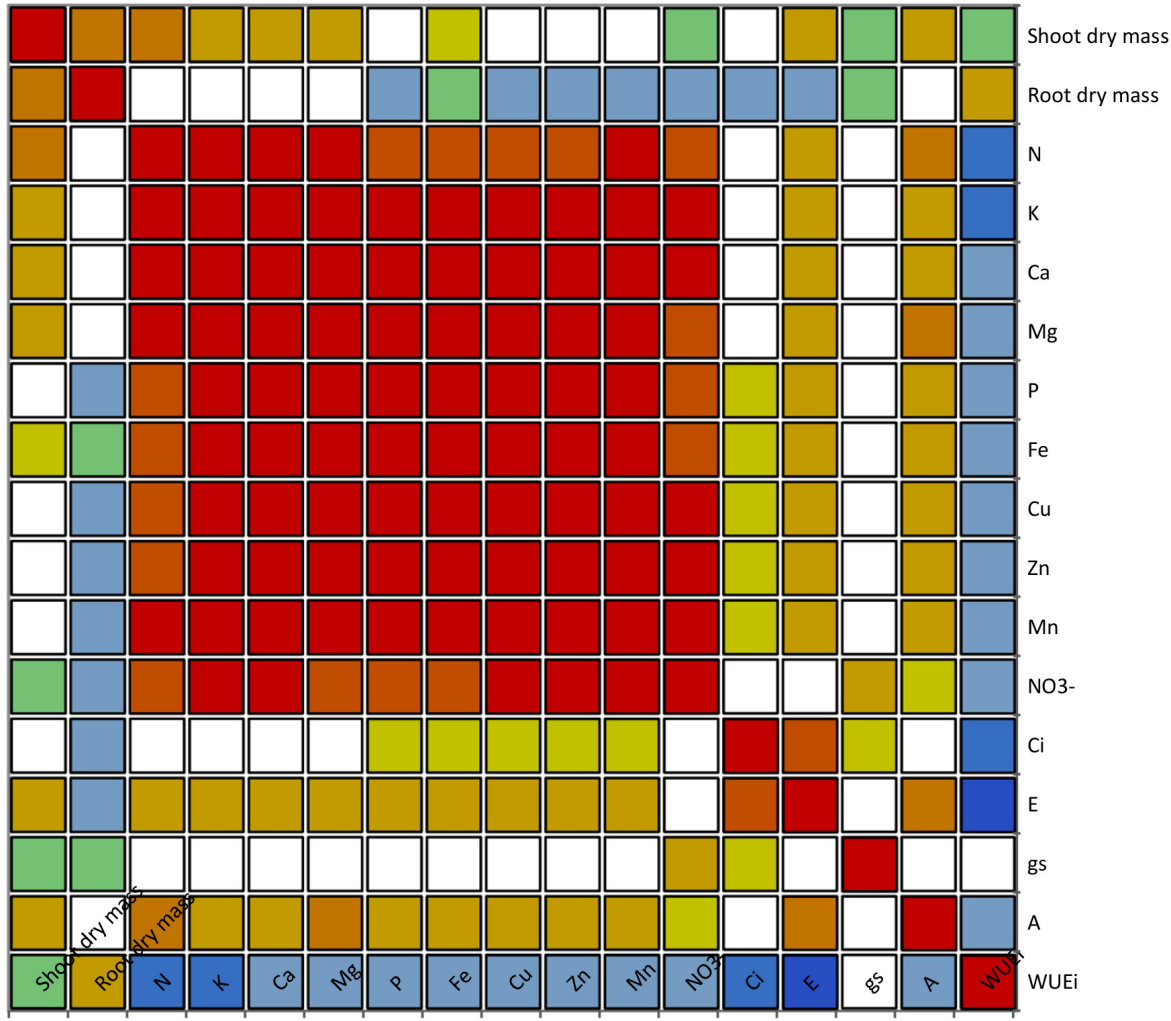
The colored correlation matrix map for obtained data under experimental treatments allowing to visually identify patterns/relationships in correlation coefficients among measured traits using a blue–red (cold‐hot) scale. The blue/cold color corresponds to a correlation near −1 (represents low correlation), and the red/hot color corresponds to a correlation near 1 (represents high correlation). Green color corresponds to a correlation near zero. Net photosynthesis rate (a), stomatal conductance (gs), intrinsic water‐use efficiency (WUEi), transpiration rate (E), and intercellular CO_2_ concentration (ci).

The net photosynthesis rate was significantly (*p* ≤ .01) affected by the employed treatments (Table 6). Here, the use of W and B LED light increased the net photosynthesis rate of LB in CR and ECBR compared to RBPN (Figure 2). But in LR, combination of red and blue LED light was more effective on net photosynthesis rate in all replacement methods of nutrient solution compared to control (plant treated with W LED and CR). Also, in LR, the use of W LED light had the lowest effect on net photosynthesis rate under CR (Table 6). Results also showed that the use of R LED light in LB had the minimum effect on net photosynthesis rate in all replacement methods of nutrient solution compared to other LED light sources (Table 6).

**TABLE 6:** The impacts of light quality and various employed methods of nutrient solution replacement on net photosynthetic rate (*P*_N_), Stomatal conductance (*g*s), and intrinsic water‐use efficiency (WUE_i_) of lettuce varieties in the NFT system.

Lettuce varieties	Light spectrum	Net photosynthetic rate (μmol m‐2 Mol‐1)			Stomatal conductance (Mol H2O m‐2 s − 1)			Intrinsic water‐use efficiency (μmol CO2 Mol H2O‐1)		
		CR	ECBR	RBPN	CR	ECBR	RBPN	CR	ECBR	RBPN
LR	W	3.13h	5.82c–e	6.8cd	0.16fg	0.25b	0.196de	19.17i	23.03hi	34.7d–f
	B	5.61d–f	5.9c–e	6.4cd	0.20de	0.20de	0.17ef	27.6f–h	29.2e–h	36.3de
	R/B	10.03ab	10.1ab	10.04ab	0.19de	0.32a	0.30a	52.9a	31.1d–h	32.7d–g
	R	6.4cd	6.17c–e	6.17c–e	0.17ef	0.24bc	0.24bc	35.58de	25.3g–i	25.5g–i
LB	W	11.18a	9.13b	6.11c–e	0.21cd	0.24bc	0.13g	52.06a	38.27cd	44.6bc
	B	7.03c	7.03c	4.49fg	0.21d	0.21d	0.16fg	33.27d–g	33.2de–g	27.6f–h
	R/B	3.63gh	6.31c–e	6.31c–e	0.08h	0.17ef	0.17ef	45.8ab	35.6d–f	36.1de
	R	4.49fg	5.11ef	5.12ef	0.16fg	0.14g	0.14g	27.6f–h	36.27de	39.3de

*Note*: Different letters indicate significant differences (*p* ≤ .05) between treatment groups according to Duncan's test.

Abbreviations: B, blue; CR, complete replacement; ECBR, EC‐based replacement; LB, Lollo Bionda; LR, Lollo Rossa; RBPN, replacing based on plant needs; R/B, Red/Blue; W, White.

### 3.14 Stomatal conductance (gs)

As shown in Table 3, stomatal conductance was significantly (*p* ≤ .01) influenced by light quality and varieties (Table 6). Also, results showed that the combination of red and blue and monochromic R LED light in LR nourishment‐based ECBR and RNPN, and white LED light spectrum in LB‐fed ECBR and CR caused an increase in stomatal conductance. Also, in LR, the use of W LED light had the lowest effect on net photosynthesis rate under CR (Table 6). Results also showed that the use of R LED light in LB had the minimum effect on net photosynthesis rate in all replacement methods of nutrient solution compared to other LED light source (Table 6).

### 3.15 Intrinsic water‐use efficiency

The Intrinsic water‐use efficiency (*WUEi*) was significantly (*p* ≤ .01) affected by the employed treatments (Table 6). Here, the use of combination of red and blue LED light in LR under CR method on nutrient solution and W and combination of red and blue LED light LB variety with CR had the highest effect on WUEi. But in LR variety, application of W LED light under CR and ECBR, and in LB variety, treatment with R LED light and CR method of nutrient solution significantly decreased the WUEi (Table 6).

### 3.16 Transpiration rate (E)

The effects of nutrient solution replacement procedures, light spectrum, varieties, and the interactions of these factors were significant on leaf transpiration rate (*p* ≤ .01, Table 7). As shown, LR variety upon exposure to monochromic R and combination r and blue LED light increased the transpiration rate by 62.3% and 62.3% in RBPN and ECBR methods compared to white light, however, other light spectra under all three employed replacement techniques of nutrient solution showed more efficient than white light spectra. In LB, the effect of blue‐light spectra under CR and ECBR methods on transpiration rate was more pronounced than white light spectra, while red‐light spectra decreased transpiration rate in plants grown under all employed replacement methods (Table 6).

**TABLE 7:** The impacts of light quality and various employed methods of nutrient solution replacement on transpiration rate (E) and intercellular CO_2_ concentration (*Ci*) of lettuce varieties in the NFT system.

Lettuce varieties	Light spectrum	Transpiration rate (Mol (H_2_O) m^−2^ s^−1^)			Intercellular CO_2_ concentration (μmol m^−2^)		
		CR	ECBR	RBPN	CR	ECBR	RBPN
LR	W	2.11h	3.75e–g	3.34g	368.6a–c	377ab	352a–d
	B	4.66b–d	4.66b–d	4.37c–e	337.3b–e	337.3b–e	319.3c–e
	R/B	3.5gh	5.36ab	4.97a–c	344b–d	334b–e	345b–d
	R	4.37c–e	5.6a	5.6a	319.3c–e	394.8a	394.a
LB	W	4.14d–f	4.93a–c	3.55gh	318.3c–e	309.6de	311.3de
	B	5.25ab	5.25ab	1.83i	383.3ab	383.2ab	303.6de
	R/B	2.38h	3.97d–g	3.82e–g	305.3de	317.6de	317.6de
	R	1.6i	1.59i	1.53i	303.6de	287.4e	287.4e

*Note*: Different letters indicate significant differences (*p* ≤ .05) between treatment groups according to Duncan's test.

Abbreviations: B, blue; CR, complete replacement; ECBR, EC‐based replacement; LB, Lollo Bionda; LR, Lollo Rossa; RBPN, replacing based on plant needs; R/B, Red/Blue; W, White.

### 3.17 Intercellular CO_2_ concentration (ci)

The *Ci* values of leaves were influenced by the employed treatments (*p* ≤ .01, Table 7). Mean analysis showed that LR variety exposed to monochromic red LED light and fortified with RBPN and ECBR methods increased the *C*_*i*_ (Table 7); but in LB variety, the use of blue LED light under CR and ECBR nutrient solution methods increased the amount of *C*_*i*_ (Table 6). However, exposure to monochromic red light in LB decreased *Ci* value compared to the control white light (Table 7).

### 3.18 Correlation and multivariate analyses

According to the correlation analysis (Figure 2), shoot dry mass was positively correlated with foliar N, K, Ca, Mg contents, net photosynthetic rate, and leaf transpiration rate. Moreover, net photosynthesis rate and leaf transpiration rate showed positive correlation with all measured minerals nutrition.

**FIGURE 3:**
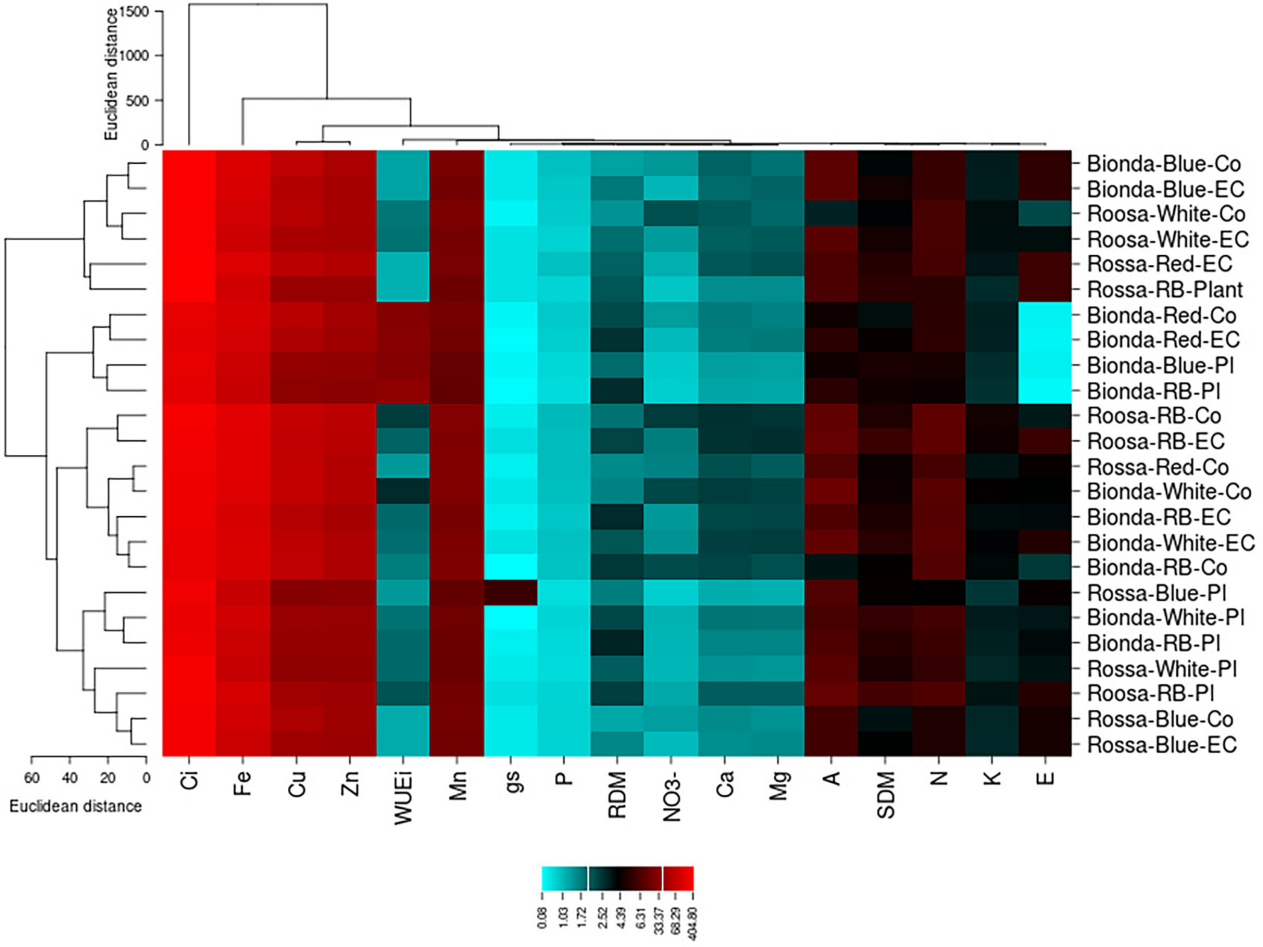
Heatmap chart shows the clustering of employed treatments in rows and measured traits in columns.

Heatmap shows the variation in measured parameters under the employed treatments by color scales, where it ranged from blue to red for the lowest and the highest values, respectively. Transpiration rate and potassium content were the most efficient parameters to reveal the changes in nutrient profile of lettuce varieties. In addition, heatmap could classify the treatments, where two distinguished groups (clusters) were obtained for the employed treatments. As shown in Figure 3, almost all treatments containing nutrient solution replacement based on plant needs were placed in a cluster.

**FIGURE 4:**
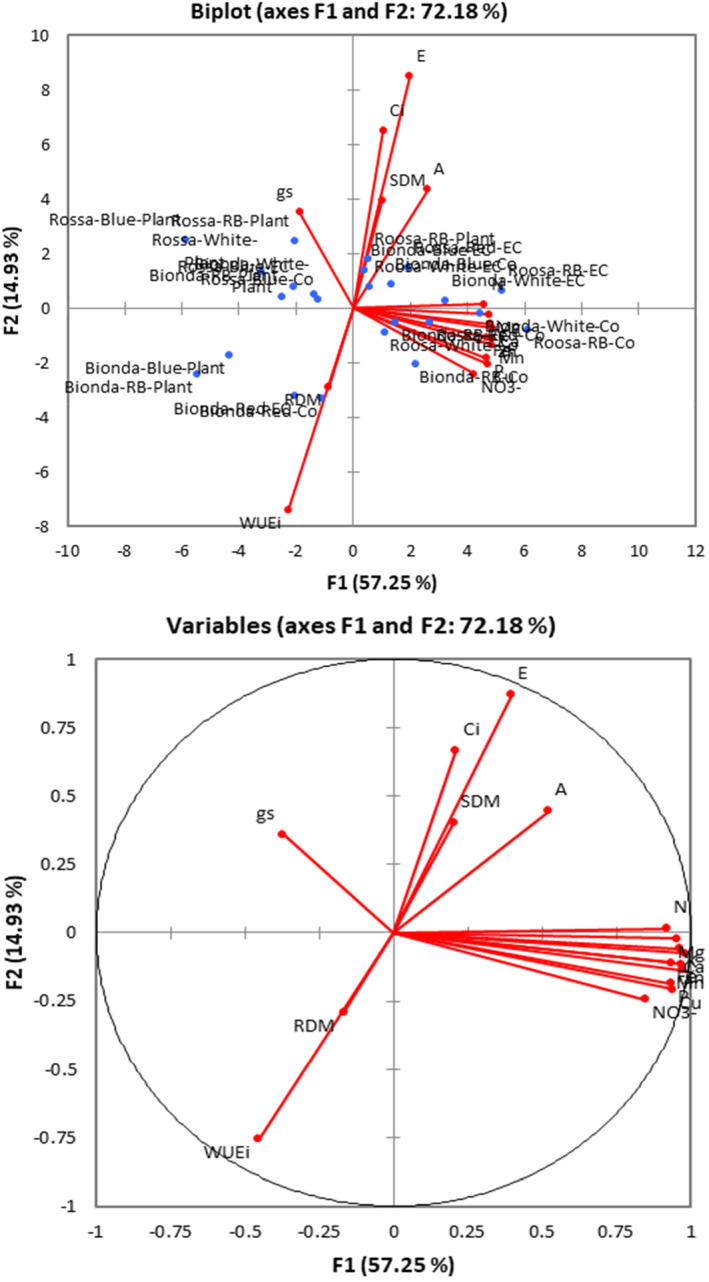
Biplot of the principal component analysis (PCA) for lettuce variety traits under experimental treatments.

Based on cluster analysis, treatment is classified into three groups. The results showed that there are 35% and 65% variations within and between clusters. Except for a treatment (Rossa‐RB‐ Plant), all treatments containing nutrient solution replacement based on plant needs were located in the second group (Figure 4).

**FIGURE 5:**
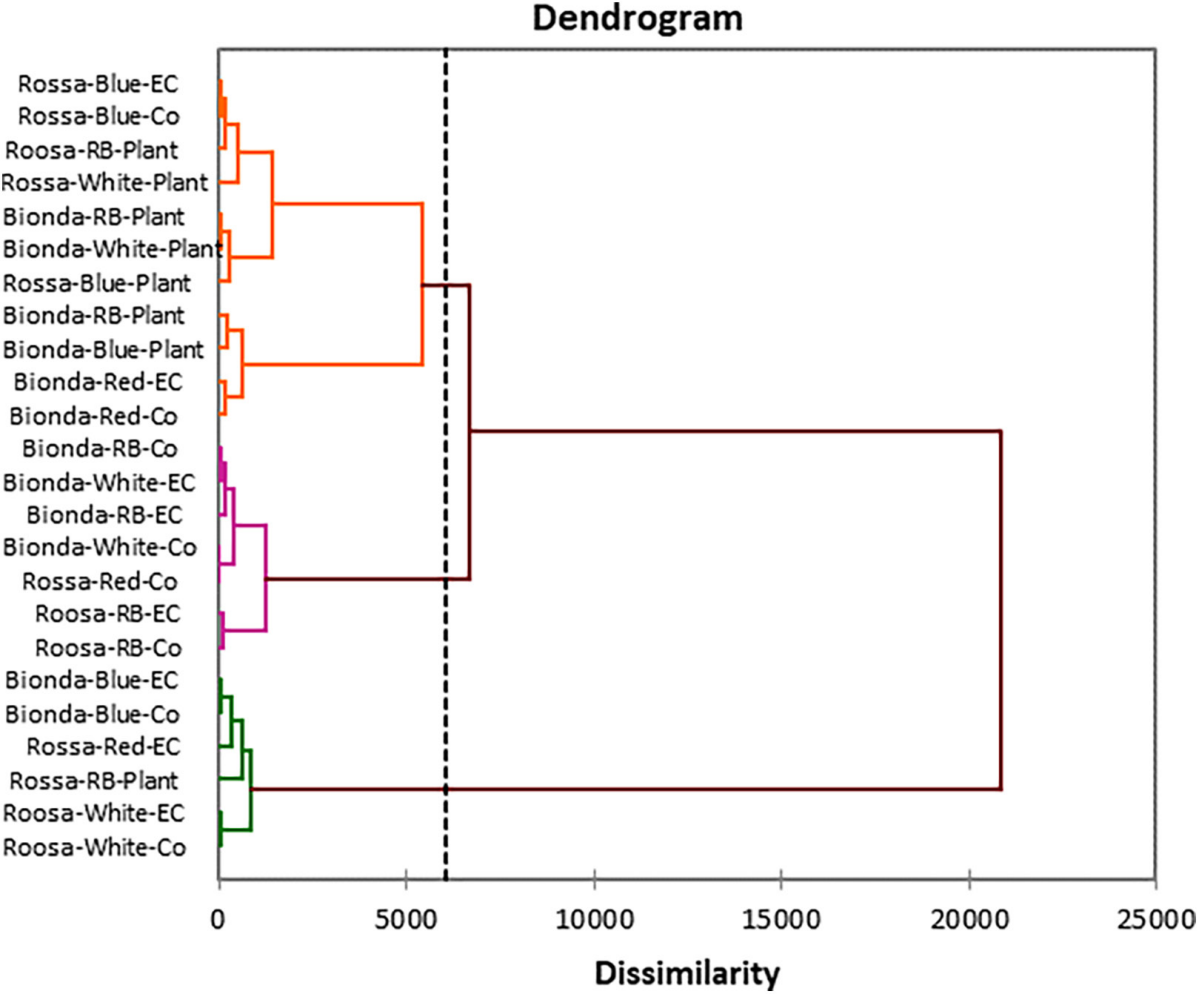
Agglomerative hierarchical clustering (AHC) dendrogram shows the progressive grouping of the obtained data under experimental treatments.

**FIGURE 6:**
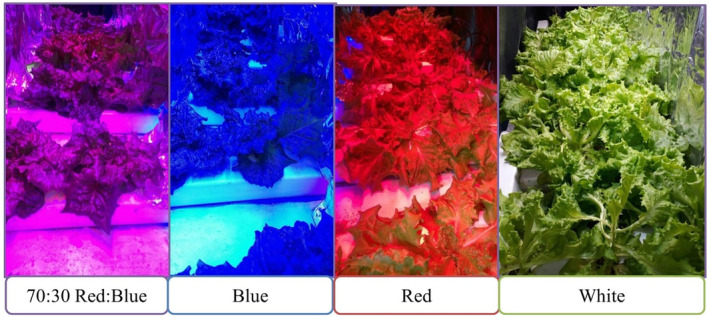
Lettuce exposure to different LED spectra under NFT system used in this study.

PCA analysis revealed that first two PCAs describe 72% of variability, while the first PCA solely describes 57% of variations and contains all mineral nutrients, nitrate, and net photosynthetic rate. Biplot showed the importance of mineral nutrients in classifying treatments (Figure 5). The general overview of lettuce plants exposed to different LED spectra under NFT system used in this study is represented in Figure 6.

## 4 DISCUSSION

In hydroponic systems, soluble form of nutrients is provided to plants. Total nutrient concentration, the elements concentration in nutrient solution, and the pH of nutrient solution are used for calculation of the nutrient solution formula (Langenfeld et al., 2022). The nutrient solution formula should also be calculated according to the plant species, type of variety, growth stage, irrigation system, and the environmental conditions (CO_2_ concentration, light, heat, and humidity) (Fussy & Papenbrock, 2022). Considering all the above mentioned, calculation of the nutrient solution formula is very complicated and requires high technical knowledge and a complex algorithm (Bevan et al., 2021; Roosta et al., 2020).

Due to the different demands of plants for nutrients in various stages of growth and development, it is necessary to change the concentration of nutrients during the growth period, although the response of cultivars to the changes will be different (Kaya et al., 2001). Changes in the concentration of nutrients led to changes in EC and pH of the nutrient solution, which further influence on absorption of nutrients by plants and ultimately affect plant growth (Smith & Roberts, 1993). It has been acknowledged that changes in nutrient solution concentration in hydroponic system cause a change in the concentration of the nutrients in corn plant tissues, and with an increase in concentration of the nutrient elements, the root activity of the plants was decreased (Smith & Roberts, 1993). Roosta et al. (2020) evaluated the pepper plants grown under different replacement techniques of nutrient solution and found that the concentrations of K, Mg, Ca, and P nutrients were affected by the replacement techniques of nutrient solution, so the maximum amount of these elements was observed in the CR method, however, the lowest concentration of these elements was observed in plant fed with ECBR. In the present study, lettuce varieties grown under RBPN showed the highest amount of N, Ca, Mg, K, and P, while application of ECBR and CR methods of nutrient solution due to the accumulation of nutrients led to imbalance of nutrient elements, and cause reduction in concentration of macronutrients (N, Ca, Mg, K, and P). The decrease in the concentration of nutrients such as N, K, and Ca under the conditions of salinity stress can be attributed to the decrease in root activity and the decrease in the absorption of water and nutrients because absorption of nutrients can be significantly reduced under such conditions (Acosta‐Motos et al., 2017; Kaya et al., 2001).

Increasing the electrical conductivity (EC) of the nutrient solution due to the addition of minerals causes a decrease in growth characteristics and a decrease in the concentration of nutrients in tomato plants (Rosadi et al., 2014). Changes in concentration of nutrients can alter the EC and pH of the nutrient solution, which reduce the osmotic potential of the solution and ultimately decrease the absorption of water and nutrients in rose ornamental plant (Kim et al., 2005). Decreasing the amount of nutrient elements in plant ECBR and CR methods of nutrient solution can be changed by adding some nutrients and disrupting the balance of nutrients due to the different absorption of elements by plants and accumulation of specific elements such as Mg. According to Regagba et al. (2014), increasing the Mg concentration of the nutrient solution caused an increase in Mg content of strawberries. Optimization of EC of nutrient solution can increase the amount of N and K, but the concentration of Mg and P remained unchanged, and the concentration of Ca was decreased (Lee et al., 2022). In the current study, combination of red and blue LED light showed more positive effect on macronutrients such as N, Ca, Mg, K, and P in LR lettuce variety. The reason for the increase in nutrients under the influence of different light spectrums can be explained by the fact that manipulation of the LED light spectrum in the conditions of optimizing the nutrient solution and preventing stress (salinity stress caused by the accumulation of nutrients in the nutrient solution) for plants may increase the activity of the photosynthetic apparatus because most of the macro‐ and micronutrient elements play an essential role in the photosynthetic apparatus (Oivukkamäki et al., 2023). Moreover, the biosynthesized carbohydrates are also transferred to the sink tissues such as roots and leaves, and in this way, the necessary energy is provided for the roots and lateral roots which is an important factor influencing water and nutrient absorption. Light indicators (e.g., light wavelength, light intensity, and duration) have a direct effect on the uptake of nutrients in plants, thereby increasing production and increasing the efficiency of nutrients that are used (Xu et al., 2021). Several studies showed that there is a close relationship between light absorption and nutrient uptake in plants (Cui et al., 2019; Sakuraba et al., 2018; Xu et al., 2021; Zhai et al., 2019). Also, researchers found that manipulation of the LED illumination spectrum can increase the absorption of macro‐ and micronutrient elements in plants (Amoozgar et al., 2017; Clavijo‐Herrera et al., 2018; Kopsell & Sams, 2013; Pennisi et al., 2019; Pinho et al., 2017).

To grow the lettuce and increase its nutritional value, the presence of optimal amount of nutrients is required (Ćavar Zeljković et al., 2023). Among the factors affecting the amount of nutrients in the leaves of leafy vegetables like lettuce, the root system that takes a role in the uptake and transfer of nutrients to the aerial organs is very important (De Pinheiro Henriques & Marcelis, 2000). In the current study, combination of red and blue LED light and replacement methods in terms of plant requirements increased the value of root dry mass. The increase in fresh and dry weight of roots and shoots of basil varieties can be justified under the influence of the combination of red and blue light and manipulation of nutrient solution that can cause an increase in the absorption of nutrients by the plant. Also, it has been determined that most nutrients play an effective role in photosynthesis and the production of primary and secondary metabolites in plants, and the increase in photosynthetic substances and their transfer to plant organs such as roots can improve the growth characteristics of plants (Roosta et al., 2020). In addition, it can be stated that genetics plays an important role in the absorption of nutrients in plants (Barker & Pilbeam, 2015). Here, the amount of fresh and dry mass of shoot and root was higher in LB variety than those of LR. One of the main reasons to increase the growth, development, and performance of plants are micronutrient elements, which is of particular importance to investigate the concentration of these elements in different conditions of plant growth and development (Roosta et al., 2020). Results of current experiment showed that LR variety fed with RBPN had the highest amount of Fe, Zn, Cu, and Mn. It has previously been reported that the use of RBPN and ECBR methods caused a decrease in concentration of nutrients in pepper plant tissues (Roosta et al., 2020), but in the present study, the concentration of Mn, Fe, Cu, and Zn nutrients was decreased in foliar parts of lettuce plants fed with ECBR and CR methods. The increase in the electrical conductivity of the nutrient solution caused by the accumulation of macronutrients such as sulfates and phosphates and the creation of salinity stress by affecting the osmotic pressure and water potential are obstacle in the direction of the transfer of water and nutrients to the roots. But in the method of feeding based on the plant needs, all nutrients were available to the plants based on the growth period of the plants, and in this method, there was no change in the EC and pH of the nutrient solution, which would cause a negative effect on the varieties of lettuce under different light treatments. Researchers believe that progressive accumulation of sulfates in nutrient solutions can limit the availability of other nutrients (Kowalska, 2004). In another study, different concentrations of nutrient solution (25%, 50%, 75%, and 100% concentrations of Hoagland's solution) on rose plants were assessed under hydroponic system, and results showed that with an increase in concentration of mineral elements in Hoagland's nutrient solution, the amount of leaf Fe also increased, and in the condition of 25% concentration of Hoagland solution, the plants showed symptoms of Fe deficiency (Zheng et al., 2010). It has been shown that increasing the EC of nutrient solution caused a significant reduction in Fe and Mn contents, and decrease in growth and osmotic potential around the root, which could be the main reason for reducing the absorption of nutrients (Calori et al., 2017). Also, previous studies showed that the combined light of red and blue increased the amount of micronutrient elements in lettuce plants (Amoozgar et al., 2017) and broccoli (Kopsell & Sams, 2013). In addition, a decrease in the amount of Fe in the leaves of plants fed with ECBR and CR methods can be due to the high pH of the nutrient solution under such conditions compared to the conditions of RBPN. So, the pH of the nutrient solution during the growth period was recorded as 7.75, 7.5, and 6.99 in the CR, ECBR, and RBPN, respectively. Since the maximum absorption of micronutrients such as iron, zinc, manganese, copper, and boron occurs in the range of 5.5 to 7, increasing the pH can cause a decrease in the absorption of these nutrients by the roots. As the researchers found that the increase in pH caused a decrease in the absorption of micronutrients such as zinc, manganese, and iron in plant species (Fageria & Zimmermann, 1998), which is consistent with the results of the present study. However, the main effects of the pH on mineral nutrition of plants occur through modification of the nutrient availability in soil (Barrow & Hartemink, 2023; Custos et al., 2020).

Accumulation of the high level of NO_3_^−^ in many leafy vegetables (e.g., lettuce, spinach, marjoram, green onion, etc.) has been previously reported (Hord et al., 2009; Premuzic et al., 2001; Sinha & Khare, 2017). Due to the environmental conditions, especially through lighting and fertilizer application, lettuce shows a high NO_3_^−^ accumulation in its leaves (Guadagnin et al., 2005; Santamaria, 2006). According to Fallovo et al. (2009), NO_3_^−^ content in lettuce leaves increased with an increase in concentration of nutrient solution. NO_3_^−^ alone is a nontoxic compound for the body (Mensinga et al., 2003; Speijers, 1996), but when approximately 5% of NO_3_^−^ combines with saliva and the digestive tract, it becomes a toxic nitrite compound (Pannala et al., 2003; Spiegelhalder et al., 1976). Vegetables contain high amounts of nutrients, phytochemicals, minerals, and vitamins, but low level of NO_3_^−^ in their tissues is one of the main factors for the loss of nutritional quality of these plants (Kosson et al., 2017). In the present study, foliar NO_3_^−^ content of lettuce varieties under RBPN and ECBR methods was decreased compared to CR procedure. Also, the combined light of red and blue, monochromic red LED light in both varieties caused the lowest accumulation of NO_3_^−^ in the plants. Photosynthetic organic compounds are made continuously under light conditions (Champigny, 1995), while NO_3_^−^ are stored in vacuoles during the night (Cheung et al., 2014). When the cells of a plant have active photosynthesis, the NO_3_^−^ stored in the vacuoles is released into the cytosol and then decomposed by nitrate reductase enzyme (Martinoia et al., 1981). The use of artificial lights on plants in the form of a combination of green light with red/blue light caused a decrease in NO_3_^−^ accumulation (Ohashi‐Kaneko et al., 2007; Velez‐Ramirez et al., 2011; Wanlai et al., 2013). It has been acknowledged that NO_3_^−^ concentration increases under blue‐light treatment (Ohashi‐Kaneko et al., 2006). In another study, it was found that NO_3_^−^ content can be reduced under blue light and upon combination of red and blue lights as compared to white light in lettuce leaves (Ohashi‐Kaneko et al., 2007). The combination of red, blue, and white lights caused a decrease in NO_3_^−^ content compared to blue light applied to lettuce plants grown in hydroponic conditions (Lin et al., 2013). It has also been reported that 1:8 ratio of blue and red light is the best light condition to reduce NO_3_^−^ accumulation in hydroponically grown lettuce plants (Jing et al., 2009; Urbonavičiūtė et al., 2009). In addition, Wanlai et al. (2013) reported that the 1:4 ratio of blue and red light caused a significant decrease in NO_3_^−^ content in lettuce. It has been found that blue and red lights have a positive role in the accumulation of carbohydrates in plants (Matsuda et al., 2004; Yorio et al., 2001). Carbohydrates can provide the necessary energy for N and NO_3_^−^ metabolism in plants (Champigny, 1995). Exposure to combination of red and blue lights with a photosynthetic photon flux density of about 200 μmol m^−2^ s^−1^ is required to reduce NO_3_^−^ accumulation and increase the quality product of lettuce (Bian et al., 2016; Chen et al., 2017). In the present study, exposure to white and monochromic LED lights (with PPFD 215 μmol m^−2^ s^−1^) under CR method increased the amount of foliar NO_3_^−^ compared to the RBPN and ECBR methods of applying nutrient solution to lettuce varieties. According to Jing et al. (2009) and Wanlai et al. (2013), the combined light of red and blue, and red LED decreased the amount of NO_3_^−^ in lettuce because these light spectrums can increase the activity of nitrate reductase in plant cells (Lillo & Appenroth, 2001) that it is contrary to the results of the present research; while in other study showed that blue light can increase NO_3_^−^ content in exposed plants (Ohashi‐Kaneko et al., 2006), which is consistent with the findings of present work. The activates of nitrate reductase and nitrite reductase in cytosol cause the conversion of NO_3_^−^ to NO_2_^−^ and ammonia (NH_4_^+^), then into glutamine or glutamate acid by the enzymes glutamine synthetase (E.C. 6.3.1.2) or glutamate dehydrogenase (EC.1.4.1.2), and finally, into amino acid, protein, and other organic biological N compounds (Meyer & Stitt, 2001; Shilpha et al., 2023).

Light is an important environmental parameter affecting stomatal conductance (Kang et al., 2016; Wang et al., 2016). The combined light of red (peaks at 634–665 nm) and blue (451 nm) are effective and useful sources of leaf photosynthesis and plant growth (Brechner & Both, 2014; Roni et al., 2017), which are widely used in commercial horticulture (Miao et al., 2016). It has been found that more than 90% of the blue‐ and red‐light spectrum (LEDs) was absorbed by the exposed plants (Gao et al., 2022). As it was found in the present study, the stomatal conductance and the rate of net photosynthesis under the treatment with a combination of red and blue LED light were increased in the RBPN and ECBR of nutrient solution (Table 6). The low values of photosynthetic characteristics of plants exposed to red LED light were due to deficiency or lack of nitrogen in plant leaves; as a result, the lower the leaf N content, the lower the content of chlorophyll and carotenoid (Kim et al., 2004). Moreover, efficiency of combined application of red and blue LED light on photosynthetic rate, leaf transpiration, and stomatal conductance in lettuce plants was recently reported by Ahmed et al. (2022) which agreed with our results. Blue‐light‐induced stomatal opening is mediated through activation of a plasma membrane (PM) H+ pump, later identified as the PM H+‐ATPase, in guard cells (Assmann et al., 1985; Matthews et al., 2020; Shimazaki et al., 2007). The blue‐light‐activated pump provides driving force for stomatal opening concomitant with ion accumulation and cell volume increase in guard cells (Kinoshita & Hayashi, 2011). Note that stomatal opening in response to weak blue light as a signal requires background red light, indicating that red light has a synergistic effect on the blue‐light response in guard cells (Inoue & Kinoshita, 2017; Shimazaki et al., 2007). However, specific mechanisms of this influence are not fully clear and can be the basis for development of new methods (Sukhova et al., 2022). Researchers suggest that the early vascular plants respond to both RL and BL and actively regulate stomatal aperture. Also, this plant absolutely requires blue light for both stomatal opening and photosynthetic CO_2_ fixation (Doi et al., 2015). Also, red light plays a critical role in stomata opening through photosynthesis in the mesophyll and guard cells of the leaves and decreases the intercellular CO_2_ concentration (Driesen et al., 2020; Shimazaki et al., 2007; Wang et al., 2016). Furthermore, Hogewoning et al. (2010) reported that blue and red light plays an essential role in chlorophyll biosynthesis. Exposure of strawberries to blue‐light, white‐light, and a combination of red‐ and blue‐light wavelengths significantly affected *Ci*, *WUEi*, and instantaneous carboxylation efficiency (*CEi*) index (Miao et al., 2016) is consistent with the results of the present research. Mixing red and blue LED light was found to be more efficient in improving photosynthesis process of lettuce plants compared to the red LED or blue LED alone (He et al., 2019). It has also been observed that exposure of lettuce plant to blue light reveals a positive impact on well‐organized guard cells with open stomata (Zheng & Van Labeke, 2017). In plants, stomata are vital channels for all gas diffusion and regulate water evaporation and CO_2_ assimilation between the plant and external environment; therefore, stomatal conductance and proton‐motive force (*pmf*) generated during photosynthesis are affected by light exposure (He et al., 2019). One of the main reasons for the decrease in transpiration rate (*E*) and leaf stomatal conductance (*gs*) in lettuce plants could be ascribed to the closure of stomata with normalized relative expression levels and the number of stomata per leaf (Muneer et al., 2014).

In our results, it was found that the combination of red and blue light in LR variety in all three replacement methods of nutrient solution increased the rate of net photosynthesis compared to the control treatment (white light and complete replacement of nutrient solution and blue light under RBPN and ECBR) (Table 6). It was also found in Table 7 that the use of blue light in RBPN and ECBR and white light in the method of CR of nutrient solution of LR variety had the highest intercellular CO_2_ concentration, but the intercellular CO_2_ concentration in the combination of red and blue light in LR variety, in all three replacement methods of nutrient solution, showed a decrease compared to the control treatment. According to the results of the net photosynthesis rate and intercellular CO_2_ concentration under red light in LB variety, the results indicate the reduction in intercellular CO_2_ concentration in the RBPN and ECBR, which have higher net photosynthesis rate compared to complete replacement of the nutrient solution (Table 7). Stomatal opening and closing control CO_2_ uptake for photosynthetic carbon assimilation and water loss by regulating transpiration, and subsequently play a critical role in crop water use efficiency and productivity (Lawson & Vialet‐Chabrand, 2019). Also, canopy gas exchange and photosynthetic traits provide insights into the energy balance of production in plants. Leaf stomatal conductance, transpiration rate, *Ci*, chlorophyll, and N contents are especially interesting characteristics because they can be determined directly on the living organs in a nondestructive manner (Parolin et al., 2010). According to Doheny‐Adams et al. (2012), a strong negative correlation was found between size and stomatal density in plants (i.e., lower stomatal density corresponds to greater leaf size). A positive correlation was also observed between steady‐state chlorophyll fluorescence and photosynthesis of plants (van der Tol et al., 2009). Moreover, a significant relationship was found between net photosynthesis and steady‐state fluorescence upon exposure to natural irradiance (Zarco‐Tejada et al., 2013). According to the research reports mentioned above, any factor that increases mineral nutrient acquisition in plants may positively affect cellular metabolism through a series of physiological and biochemical activities.

## 5 CONCLUSIONS

This research was conducted in order to investigate the effects of different LED spectrums on morpho‐physiological characteristics and nutritional values of two lettuce varieties fed based on different replacement methods of nutrient solution. Results showed that manipulation of LED spectrums along with plant nourishment based on the plant needs in both varieties positively affects net photosynthesis rate, stomatal conductance, transpiration rate, intercellular CO_2_ concentration of leaves, intrinsic water‐use efficiency and absorption, and transfer of various essential elements including macro‐ and micronutrient that play a critical role in the growth and development processes. Due to the timely and required adjustment of elements in the nutrient solution, RBPN and ECBR techniques provide better control of water and nutrient elements consumption than CR method to achieve high production efficiency with lower cost and without harming the environment. Also, such replacing methods can be easily used in all regions of the world that face a wide array of environmental challenges.

## AUTHOR CONTRIBUTIONS

**Hamid Reza Soufi:** Conceptualization (equal); data curation (equal); formal analysis (equal); writing – review and editing (equal). **Hamid Reza Roosta:** Data curation (equal); funding acquisition (equal); methodology (equal); supervision (equal); validation (equal); writing – review and editing (equal). **Foad Fatehi:** Data curation (equal); formal analysis (equal); visualization (equal); writing – original draft (equal). **Mansour Ghorbanpour:** Formal analysis (equal); methodology (equal); validation (equal); writing – review and editing (equal).

## CONFLICT OF INTEREST STATEMENT

The authors declare no conflict of interest.

## ETHICS STATEMENT

This material is the authors’ own original work, which has not been previously published elsewhere. Also, the manuscript is not currently being considered for publication elsewhere. This manuscript reflects the authors' own research and analysis in a truthful and complete manner.

## CONSENT TO PARTICIPATE

All authors have been personally and actively involved in substantial work leading to the manuscript and will take public responsibility for its content.

## CONSENT FOR PUBLICATION

All authors have agreed to submit the manuscript in its current form for consideration and possible publication in *Food Science & Nutrition*.

## Data Availability

All data generated or analyzed during this study are included in this published article.
